# In-bed cycling for adult intensive care patients: A systematic review and meta-analysis

**DOI:** 10.1097/MD.0000000000047608

**Published:** 2026-02-20

**Authors:** Deping Lü, Xihui Sun, Min Feng, Ping Zhao, Huineng Xiao

**Affiliations:** a Operating Theatre, Beijing Anzhen Nanchong Hospital of Capital Medical University & Nanchong Central Hospital, Nanchong, Sichuan, China; b Department of Nursing Science, Faculty of Medicine, Universiti Malaya, Kuala Lumpur, Wilayah Persekutuan Kuala Lumpuri, Malaysia; c Nursing Department, Bishan hospital of Chongqing Medical University (Chongqing Bishan District People’s Hospital), Chongqing, China; d Hemodialysis Department, Bishan hospital of Chongqing Medical University (Chongqing Bishan District People’s Hospital), Chongqing, China; e Affiliated Hospital of North Sichuan Medical College, Nanchong, China.

**Keywords:** delirium, ICU patients, ICU-AW, in-bed cycling, meta-analysis, muscle strength

## Abstract

**Background:**

Integrating in-bed cycling (IBC) into graded early mobilization is common in intensive care units (ICUs), yet findings remain mixed on whether it yields significant improvements in muscle strength, mechanical ventilation outcomes, and ICU complications.

**Methods:**

We conducted a comprehensive search for literature published from database construction to July 31, 2025. The searched databases include PubMed, Cumulative Index to Nursing and Allied Health Literature, Web of Science, and other 8 databases. We assessed the quality of the included studies using the Cochrane Risk of Bias tool and analyzed data using RevMan 5.4 software. The Grading of Recommendations Assessment, Development and Evaluation system was used to assess the certainty of the evidence.

**Results:**

Data from 41 randomized controlled trials (n = 3781) show that adding passive/active IBC to routine ICU rehabilitation improves Medical Research Council (standardized mean difference [SMD] = 0.53; 95% confidence interval [CI] [0.01–1.05], *P* = .04), 6-minute walking distance (SMD = 0.80; 95% CI [0.14–1.45], *P* = .02), handgrip (SMD = 0.09; 95% CI [0.02–0.36], *P* = .03), and Barthel Index (SMD = 1.06; 95% CI [0.66–1.46], *P* < .001). It shortens MV time (SMD = −0.28; 95% CI [−0.47 to −0.10], *P* = .001), ICU stay (SMD = −0.32, 95% CI [−0.53 to −0.12], *P* = .002), and hospital stay (SMD = −0.22, 95% CI [−0.40 to −0.03], *P* = .02), and lowers ICU-acquired weakness (odds ratio [OR] = 0.49, 95% CI [0.37–0.67], *P* < .001) and delirium (OR = 0.50, 95% CI [0.33–0.77], *P* = .002). ICU mortality (OR = 0.95, 95% CI [0.76–1.19], *P* = .65) and exercise-related adverse events (OR = 0.77, 95% CI [0.53–1.14], *P* = .20) were not increased. Results were stable in sensitivity analyses, with very low-to-moderate certainty.

**Conclusion:**

Adjunct IBC alongside routine ICU rehabilitation is associated with clinically meaningful gains in strength and functional recovery, shorter dependence on organ support and ICU stay, and lower neuromuscular and neurocognitive complications, without an evident safety penalty. These effects were robust to sensitivity analyses, though the overall certainty of evidence is very low to moderate, underscoring the need for large, well-designed trials with standardized IBC protocols, longer-term outcomes and economic evaluation.

## 1. Introduction

Survivors of the intensive care unit (ICU) often endure lasting organ damage and functional impairments, which adversely affect their cognitive function and overall.^[1]^ This vulnerability to a range of complications, including pneumonia, pressure ulcers, acquired weakness in the ICU (ICU-AW), lower limb thrombosis, and more, is attributed to systemic inflammation, hyperglycemia, poor nutritional absorption, prolonged immobility, and the use of sedative and analgesic medications.^[2,3]^ Notably, ICU-AW and delirium afflict up to 75% and 40% of patients, respectively, significantly impacting their quality of life and long-term physical function.^[4,5]^

In response to these challenges, early mobilization strategies have been incorporated into ICU patient rehabilitation, aiming to prevent muscle atrophy, enhance muscle strength, and expedite ventilator weaning. Such strategies include limb exercises, posture training, neuromuscular electrical stimulation, and in-bed cycling (IBC).^[6–8]^ IBC, whether used alone or in conjunction with other physical exercises, has emerged as a promising intervention, advocated by healthcare professionals and researchers for its potential to maintain cross-sectional muscle area, muscle activity, and promote blood flow to prevent lower extremity thrombosis.^[9–12]^ The safety and feasibility of IBC in ICU patients have been documented, alongside several potential benefits such as improved motor function, reduced muscle loss, lower delirium incidence, and shorter durations of mechanical ventilation (MV) or ICU stay.^[13–16]^

However, previous studies demonstrate heterogeneous findings regarding the benefits of IBC exercise for ICU patients. For instance, a Belgian study observed that 20 minutes of daily passive IBC exercise-maintained diaphragm cross-sectional area in ICU septic patients but did not shorten MV or hospital stays.^[17]^ A Swiss study indicated that daily passive or active IBC may influence psychological scores post-discharge but does not effectively enhance physical function or muscle strength upon ICU discharge.^[18]^ Additionally, a high-quality, large-sample single-center randomized controlled trial (RCT) revealed that lower limb muscle electrical stimulation combined with an hour of daily IBC exercise did not alter overall muscle strength or delirium incidence at discharge or 6 months later, complicating the assessment of IBC’s specific impact.^[11]^ Australian scholars implemented usual care-based IBC exercise for ICU patients, instructing them to engage in active exercise for 30 minutes daily. Their results suggested that the muscle atrophy area showed no significant difference between the experimental and control groups.^[19]^ A recent large Canadian RCT likewise found no significant improvements in physical function or quality of life at ICU discharge beyond routine rehabilitation,^[20]^ whereas other studies report that IBC reduces ICU-acquired weakness and improves 6-minute walk distance (6MWD).^[14,21]^

Furthermore, 2 other studies explored the effects of neuromuscular electrical stimulation combined with IBC exercise on ICU patients. They found that 60 to 90 minutes of IBC exercise combined with electrical muscle stimulation had no significant impact on lower limb muscle cross-sectional area at discharge, the patient’s physical function 6 months after discharge, MV duration, or delirium incidence.^[9,22]^ The varied conclusions across these studies underscore the need for more robust evidence to reevaluate IBC’s true impact on MV outcomes, muscle strength, and cognitive status in ICU patients. This necessity paves the way for our research, aiming to provide substantial guidance for effective clinical practice and targeted future research efforts, which could help optimize resource allocation and guide future research priorities.

In summary, the influence of IBC on muscle strength, delirium, and other clinical outcomes in ICU patients remains uncertain. The existing research, fraught with divergent results and a scarcity of high-quality, evidence-based studies, leaves healthcare professionals in a quandary over the utilization of IBC in the ICU setting. Addressing this gap, this study seeks to elucidate the precise efficacy of IBC through rigorous meta-analysis and systematic review, aiming to furnish the robust evidence base needed to guide clinical practice confidently.

## 2. Methods

### 2.1. Aims

This paper aims to systematically evaluate the influence of IBC on various indicators among ICU patients, based on original data from included RCTs. The primary outcome measures for this meta-analysis are the Medical Research Council (MRC) score, 6MWD, hand grip strength, activities of daily living (ADL) score, incidence of ICU-AW and delirium. Secondary outcomes include ICU mortality, MV time (MVT), ICU length of stay (ICU-LOS), length of hospital stay, and adverse events.

### 2.2. Design

This study constitutes a meta-analysis and systematic review of prospective RCT studies, conducted in accordance with the Preferred Reporting Items for Systematic Reviews and Meta-Analyses (PRISMA) recommendations^[23]^ and registered with International Prospective Register of Systematic Reviews (CRD42023475722).

### 2.3. Search strategy

The literature search, spanning from Database construction to July 31, 2025, was independently conducted by 2 researchers (HN and DP). Primary databases searched included PubMed, Scopus, Web of Science, Wiley Online Library, ScienceDirect, Cochrane Library, Cumulative Index to Nursing and Allied Health Literature, and China National Knowledge Infrastructure (CNKI). The search used a mix of subject-specific and free-text Mesh terms. Keywords for the search included: “intensive care unit,” “ICU,” “critically ill patients,” “mechanically ventilated patients”, “mechanical ventilation patients,” “intensive care patients,” “in-bed cycling,” “early exercise,” “early rehabilitation,” “in-bed cycle ergometry,” “cycling exercise,” “bed bicycle exercise,” “bedside Cycling,” “Early mobilization,” “bed bicycle exercise,” among others. Additionally, references of included high-quality studies closely related to the research topic were meticulously reviewed.

### 2.4. Eligibility criteria

Screening of titles, abstracts, and full texts of the retrieved articles was performed by 2 reviewers, SX and MJ, based on the following inclusion criteria:

- Studies with RCT design, including both parallel and crossover designs.
- Adult ICU patients with a stay exceeding 48 hours.
- Studies incorporating indicators such as MRC, Barthel Index (BI), delirium incidence, handgrip strength, ICU-AW, MVT, ICU-LOS, ICU mortality, hospital stay, adverse events and 6MWD.
- Control groups receiving routine rehabilitation or usual care, involving active and passive limb mobilization.
- Experimental groups receiving passive or active IBC in addition to routine rehabilitation, with specifics of the IBC exercise tailored to the patient’s condition and willingness.

### 2.5. Exclusion criteria

Excluded were studies involving:

- Pediatric ICU participants.
- Non-RCT designs, such as cohort studies, comments, or reviews.
- Duplicate publications.
- Studies focusing primarily on the feasibility and safety of IBC.
- Outcome measures not aligning with the study’s inclusion criteria.
- Studies without available full-text or extractable data.
- Publications not in Chinese or English.

### 2.6. Study screening process

**Figure 1.**
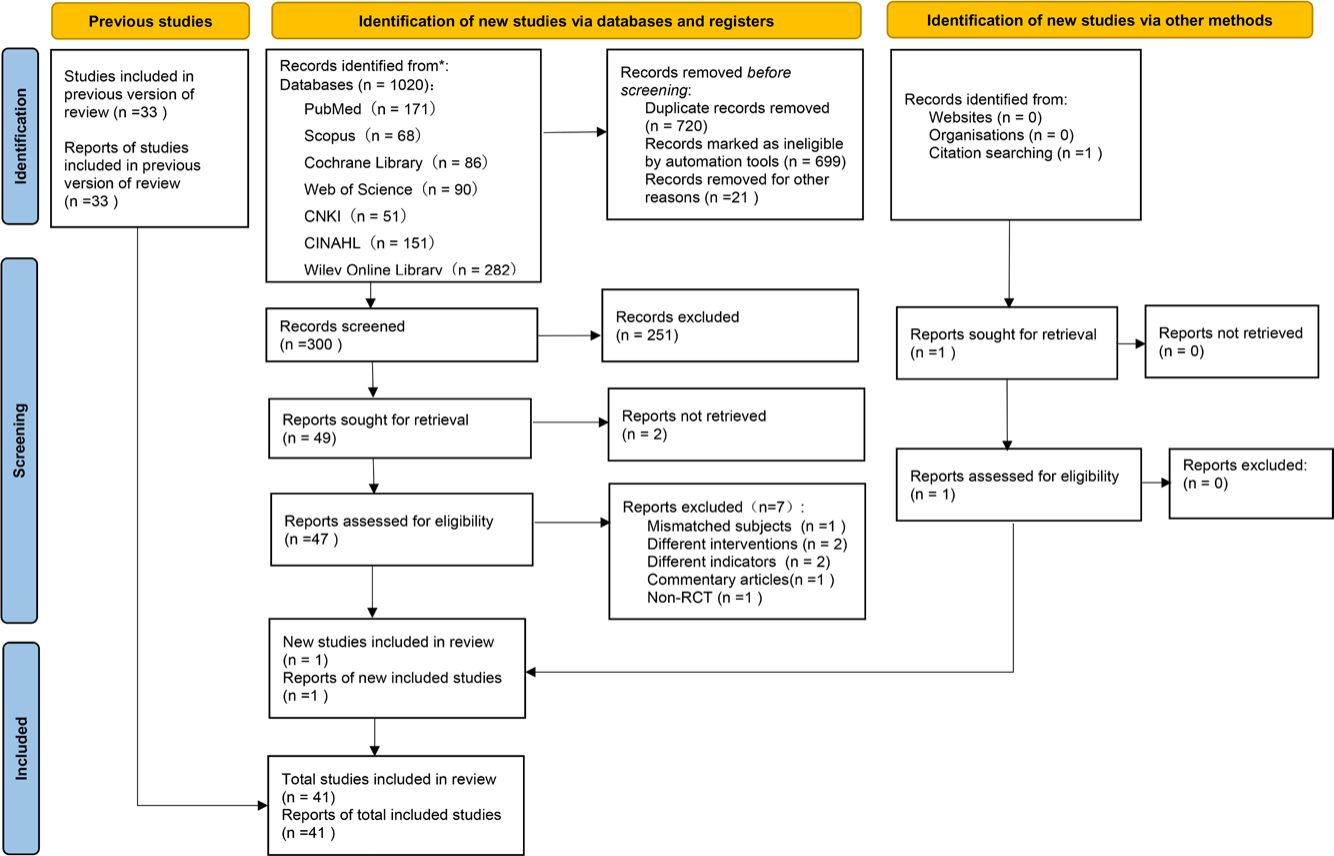
Study screening process (based on PRISMA 2020). Researchers screened and excluded literature based on PRISMA 2020. PRISMA = Preferred Reporting Items for Systematic Reviews and Meta-Analyses.

Using EndNote X9 to manage citations, initially, 1020 studies were identified through database searches. After deduplication and applying exclusion criteria, 300 studies were considered for further evaluation. A total of 251 studies were excluded for reasons such as irrelevant topic or design, leaving 49 studies. Following full-text screening, 41 studies were included, with 40 contributing to the meta-analysis. Figure 1 illustrates the literature screening process.

### 2.7. Data extraction

To ensure a thorough review, researchers ZP and SX independently screened titles and abstracts to identify studies that met the inclusion criteria. Disagreements were resolved through discussion with a third researcher. Upon selection, data were meticulously extracted into a structured table, capturing essential details such as author(s), year of publication, country of study, sample size, description of intervention (experimental and control groups), and outcome indicators. These indicators encompassed the MRC score, delirium incidence, ICU-AW rate, BI score, MV time, ICU stay, and 6MWD, among others.

### 2.8. Quality appraisal

The Cochrane Handbook for Systematic Reviews of Interventions, version 6.3^[24]^ served as the foundation for bias assessment tool, with HN and DP conducting independent reviews. Evaluation domains included the randomization process, allocation concealment, blinding of implementation, data integrity, selective reporting, and other biases.

### 2.9. Data synthesis and analysis

Meta-analysis was performed using RevMan 5.4 software (The Cochrane Collaboration, Oxford, UK) by ZP and SX. Dichotomous outcomes were analyzed using odds ratios (ORs) and their 95% confidence intervals (CIs), while continuous outcomes utilized the standardized mean difference (SMD) and 95% CIs. Heterogeneity was assessed via the *Q* test and *I*^2^ statistic, with classifications of low (<25%), medium (25–75%), or high (>75%) heterogeneity.^[25]^ Depending on the heterogeneity level, appropriate models (fixed-effects or random-effects) were selected for analysis.^[26]^ Sensitivity analyses and publication bias assessments were conducted as needed. For data presented as medians and ranges, mean and standard deviation calculations followed established statistical formulae.^[27]^

### 2.10. Clinical evidence grading

The quality of evidence for each outcome was evaluated using the Grading of Recommendations Assessment, Development and Evaluation profiler (version 3.6.1) scoring system, distinguishing high confidence estimates typically associated with RCTs from lower confidence estimates of observational studies. Evidence grading covered aspects of review bias, inconsistency, methodological limitations, imprecision, and indirectness, with levels ranging from very low to high.^[28]^

## 3. Results

### 3.1. Search summary

The initial search yielded 3 hundred articles after duplicate removal. Screening for relevance reduced this number to 47 full texts, from which 7 were excluded due to various reasons: inconsistency in study subjects (n = 1);^[29]^ commentary articles (n = 1);^[30]^ different intervention protocols (n = 2);^[31,32]^ variations in outcome measures (n = 2);^[33,34]^ non-RCT (n = 1).^[35]^ One additional eligible study was identified through other sources.^[36]^ A total of 41 RCTs were eligible for inclusion in this meta-analysis (as shown in Table 1).

**Table 1:** Basic information of studies included (N = 41).

References/country	Sample size E/C	Age E/C	Type of patients	Intervention program for IBC group			Control group	Primary outcome	Measure tool
				Start point, frequency, intensity, and duration	Follow-up	Adverse events of IBC			
Nava^[37]^, Italy	60:20	E:65 ± 6 C:67 ± 9	COPD patients on MV in RICU	Start: 2 wk post-admit Intervention plan: Morning IBC for ~20 min, titrated to avoid dyspnea.	ICU discharge	None	RR	③⑤⑦	6MWD, days
Burtin et al^[38]^, Belgium	31/36	E: 56 ± 16 C: 57 ± 17	Cardiac, respiratory or surgical patients	Start: 5 d post-admit Intervention plan: IBC, 20 r/min, 20 min/session, 5 d/wk	Hospital discharge	None	RR	②④⑤⑤⑥⑦	MRC, days 6MWD
Dantas et al^[39]^, Brazil	14/14	E: 59.07 ± 15.22 C: 50.43 ± 20.45	Diabetes mellitus, myocardial infarction.	Start: NR Intervention plan: IBC, 2 sessions/d, 7 d/wk	NR	NR	RR	②⑦	MRC, days
Kayambu et al^[40]^, Australia	26/24	E: 62.5 (30–83) C: 65.5 (37–85)	Septic patients on MV > 48 h	Start: <48 h post-admit Intervention plan: IBC + EMS, 30 min/session, 2 times/d	ICU Discharge 6 mo	NR	RR	②④⑤⑥⑦	MRC, days
Coutinho et al^[41]^, Brazil	11/14	E: 55.2 ± 29.2 C: 61.8 ± 22.1	Patients with mechanical ventilation	Start: after MV Intervention plan: IBC, 20 min/session, 1 time/d	NR	NR	RR	④⑤⑥⑦	Days,
Borges et al^[42]^, Brazil	15/19	E: 62.5 ± 7.1 C: 62.8 ± 4.2	Post-CABG patients	Start: early postoperative period Intervention plan: IBC 1–2×/d, 5–20 min, until discharge.	Hospital discharge	NR	RR	③⑤⑥⑦	6MWD Days
Machado et al^[43]^, Brazil	22/16	E: 44.46 ± 19.23 C: 45.13 ± 18.91	Nerve, respiratory, cardiac, abdominal and other diseases	Start: <72 h MV Intervention plan: passive IBC, 20 min/session, 7 d/wk,	ICU discharge	NR	RR	②⑤⑥⑦	MRC, days
França et al^[44]^, Brazil	9/10	E: 77 (32–81) C: 55 (44–70)	Respiratory/Cardiac Sepsis/infection	Start: NR Intervention plan: IBC, 30 rpm/min, 20 min/session.	NR	NR	RR	④⑤⑥⑦	Days
Bianchi et al^[10]^, Brazil	18/14	E: 52.3 ± 22.7 C: 56.1 ± 23.0	Sepsis, etc	Start: post-MV initiation Intervention plan: passive IBC, 20 r/min, 20 min/d,	At estuation/discharge	NR	RR	④⑤⑥⑦	Days
Hickmann et al^[17]^, Belgium	9/10	E: 59 ± 19 C: 57 ± 20	Sepsis shock	Start: <72 h after admission Intervention plan: Passive or active IBC, 30 min/session, 2 sessions/d, 7 times/wk	1 wk	NR	RR	②④⑤ ⑦⑩	MRC, days,
Eggmann et al^[18]^, Switzerland	58/57	E: 65 ± 15 C: 63 ± 15	Heart surgery, Neurosurgery, Respiratory insufficiency	Start:<48 h post-admission Intervention plan: Active IBC, 20 r/min, 20–30 min/session,	6 mo	4 cases: Low SpO_2_	RR	②④⑤⑥⑦⑧⑩	MRC, #ays, 6WMD
Fossat et al^[11]^, France	158/154	E: 65 ± 13 C: 66 ± 15	Severe pneumonia, respiratory failure.	Start: after random allocation Intervention plan: IBC and MES, 65 min/session, 5 d/wk	6 mo	8 cases: Treatment interruption	RR	①②④⑤⑦⑩	MRC, days, CAM-ICU,
Zhu et al^[45]^, China	45/45	E: 65.93 ± 6.18 C: 64.07 ± 6.85	COPD exacerbation	Start: NR Intervention plan: IBC, 15 min/session, 2 times/d	2 wk	NR	RR	④⑧	MRC Days
Dou et al^[46]^, China	60/60	E: 53.97 ± 4.14 C: 53.50 ± 3.56	Pneumonia, septic shock,	Start: NR Intervention plan: IBC, 15 min/session, 2 times/d	2 wk	NR	UC	①④⑤	CAM-ICU Days
Wu^[37]^, China	133/133	E: 45.99 ± 15.60 C: 44.57 ± 16.59	Adult MV patients with varied diagnoses	Start: NR Intervention plan: IBC, 30 min/session, once/day	ICU discharge	NR	RR	①④⑤	CAM-ICU Days
Ji^[12]^, China	48/48	E: 61.74 ± 13.57 C: 60.32 ± 13.14	Pneumonia, acute renal failure,	Start: NR Intervention plan: Active or passive IBC, 15–30 r/min, 20 min/2 session/d	ICU discharge	No	RR	②④⑤ ⑨⑩	Days, B-ultrasound
Carvalho^[47]^, Brazil	12/12	E: 47.83 ± 19.61 C: 54.17 ± 16.71	MV>24 h	Start: 24–48 h MV Intervention plan: IBC, 20 min/session, 1 session/d, 1 wk	1 wk	No	RR	⑨	B-ultrasound
Kho et al^[48]^, Canada	36/30	E: 60.0 ± 16.8 C: 63.6 ± 17.1	Respiratory, cardiovascular, gastrointestinal, etc	Start: within 24 h of ICU admission Intervention plan: Passive IBC, 30 r/min, 1 session/d	ICU discharge	1 case: Arrhythmia	RR	④⑤⑥⑦⑩	MRC, days
Seo et al^[49]^, Korea	8:8	E: 67.37 ± 10.82 C: 66.50 ± 8.66	ICU stay ≥ 5 d; communicative	Start: after admission Intervention plan: IBC, 30 min/session, 2 times/d	ICU discharge	None	RR	②③④⑤	MRC, days, FSS-ICU
Gama et al^[50]^, Brazil	111/117	E: 58.20 ± 12.90 C: 57.20 ± 13.20	Cardiovascular surgery	Start: day 1 after surgery Intervention plan: 2 times/d, 10 min/session, extubation to ICU discharge	ICU discharge Hospital discharge	None	RR	④⑤	Days
Yu et al^[51]^, China	52/54	E: 59.98 ± 8.01 C: 58.37 ± 7.35	Aute respiratory failure	Start: NR Intervention plan: Active or passive IBC, 15–30 min/session	ICU discharge	NR	RR	④⑤⑦ ⑧	Days, MRC
Schujmann et al^[52]^, Brazil	29:29	E: 42 (38–46) C: 43 (40–46)	severe burns	Start: within 48 h of ICU admission Intervention plan: 2 times/d, 20–30 min/session From admission to discharge	6 wk 12 wk	None	RR	③⑤⑥	Days BI
Nickels et al^[19]^, Australia	36/36	E: 56 ± 18 C: 57 ± 16	Sepsis, cardiac surgery,	Start: MV ≥ 48 h Intervention plan: IBC, 30 min/session, 1 session/d	7 d after discharge	NR	RR	②③④⑤⑥⑦⑧	MRC, FSS-ICU, CAM-ICU, Days,
Ji et al^[53]^, China	38/38	E: 46.88 ± 16.32 C: 45.51 ± 15.18	Abdominal surgery	Start: before routine rehabilitation Intervention plan: Active or passive IBC, 20 min/session, 2 times/d	At ICU discharge	No	RR	④⑤⑧	MRC Days
Berney et al^[9]^, Australia	80/82	E: 61 (51–69) C: 59 (48–67)	Sepsis, postoperative patient	Start: <24 h after in ICU Intervention plan: IBC, 60 min/d, 5 d/wk	3 mo 6 mo	NR	RR	③④⑤⑥⑦⑧	MRC, days, 6MWD, FSS-ICU
Waldauf et al^[22]^, Czech Republic	75/75	E: 59.9 ± 15.1 C: 62.3 ± 15.4	Arborescence, multiple traumas, heart failure	Start: 1 d after admission Intervention plan: Passive IBC and MES, 90 min/d, 7 d/wk	ICU discharge, 6 mo	NR	RR	②④⑤⑥	MRC Days, SF-36
Ribeiro et al^[54]^, Brazil	16:15	E: 60.30 ± 8.30 C: 58.30 ± 7.70	Coronary artery surgery	Start: NR Intervention plan: IBC, 3–20 min/time, Totally 3 times	Day 4 after surgery	None	RR	④⑤⑥⑧	MRC Days
De Azevedo et al^[55]^, Brazil	87:94	E: 6760 ± 17.8 C: 65.30 ± 19.70	ICU patients >3 d	Start: NR Intervention plan: Passive/active cycle ergometry, twice daily for 15 min until discharge	ICU discharge, 3 mo 6 mo	None	RR	④⑤⑥⑦⑧	MRC Days
Deng et al^[56]^, China	41:42	E: 5640 ± 14.93 C: 53.44 ± 16.85	Acute type A aortic dissection	Start: NR Intervention plan: IBC, 2 wk	Day 7 after implementation	1 peritoneal tube prolapse 1 hypoxemia case	RR	④⑤⑩	Days
Qie et al^[57]^, China	39:38	E: 53.87 ± 2.85 C: 52.52 ± 3.04	MV ≥ 3 d	Start: NR Intervention plan: Asymptomatic IBC, 1–2 sessions/d 30 min/session	ICU discharge	None	RR	①③④⑤⑧⑩	CAM-ICU, Days, BI
Yu et al^[58]^, China	52/50	E: 69.96 ± 8.14 C: 69.74 ± 8.13	Severe pneumonia patients on MV, ≥7-d stay.	Start: stable vital signs Intervention plan: Passive/active IBC, 5×/wk, 30 min/mission, 1 wk	ICU discharge	None	RR	③⑤⑥	Days, ADL
Bano et al^[59]^, Pakistan	51:51	E: 57.43 ± 9.15 C: 55.62 ± 7.62	Coronary surgery patients with MV < 12 h	Start: day 2 after surgery Intervention plan: Active IBC, 20–35 min/session	Day 4 after surgery	NR	RR	③④⑤⑥	6MWD, Days
Lin et al^[16]^, China	38:39	E: 52.32 ± 13.86 C: 53.44 ± 12.06	Type A aortic dissection patients	Start: <48 h post-operation Intervention plan: Active IBC and EMS, 30–50 min/session	ICU discharge, 3 month	1 agitation, And 1 BP instability	RR	②③④⑤⑥⑧⑩	BI, MRC, Days
Maca et al^[15]^, Czech Republic	20:19	E: 67.00 ± 12.59 C: 53.00 ± 16.02	MV patients	Start: post-weaning Intervention plan: Active IBC, 10 min/d, 5 d and 2 wk	ICU discharge	No	RR	②③④⑤⑦	BI, Days,
Zhou et al^[60]^, China	33/33	E: 51.89 ± 8.27 C: 52.17 ± 8.04	Postoperative pulmonary patients	Start: after assessment Intervention plan: active or passive IBC, 20 min/time, 2 times/d	2 wk	None	RR	①④⑤	CAM-ICU, Days,
Soto et al^[14]^, Chile	33:36	E: 60.0 ± 11.5 C: 591.±12.5	ICU patients requiring MV > 48 h	Start: within 48 h post-enrolment Intervention plan: Progressive IBC exercises, 45 min daily until discharge	ICU discharge	None	RR	①②③④⑤⑦⑧	BI, FFS-ICU MRC, Days
Ahmad et al^[21]^	15:16	E: 33.20 ± 6.29 C: 33.44 ± 5.77	Cardiac surgery patients, 20–40 yr	Start: postoperative day 1 Intervention plan: IBC, 15–20 min/session, 1 wk	Hospital discharge 1-month	NR	RR	③④⑤⑥	BI, Days, 6MWD
Wu (2024), China	32:32	E: 33.20 ± 6.29 C: 33.44 ± 5.77	MV patients	Start: MV day 2 Active or passive IBC, 20 min/time, 2 times/d	ICU discharge	NR	RR	①④⑤	CAM-ICU Days
Kho et al^[20]^, Canada	182:178	E: 61.20 ± 15.80 C: 61.80 ± 15.40	ICU patients with MV < 4 days	Start: MV day 2 Intervention plan: Passive/active IBC, 5×/wk, 30 min/mission, until ICU discharge	ICU and hospital discharge	9 cardiac, 8 respiratory, 1 other event	RR	②④⑤⑥⑦⑧⑩	MRC, Days
Zhang et al^[13]^, China	31:31	E: 55.10 ± 9.78 C: 55.8111.50	Liver transplant patients with extubation ≤ 48 h	Start: postoperative extubation and awakening Intervention plan: 20 min/time, once/d, until discharge from ICU	ICU discharge Hospital discharge	None	RR	⑤⑥⑦⑨⑩	BI, Days B-ultrasound
Lorenz et al^[36]^, Germany	10:10	E: 55 (47–67) C: 61 (56–68)	COVID-19 patients on MV > 24 h	Start: obtaining consent on randomization day Intervention plan: Daily 20-min robot-assisted in-bed cycling.	ICU discharge	None	RR	③⑨⑥⑩	IOS, SOMS B-ultrasound

*Note*: Outcome: ① delirium rate; ② muscle strength; ③ physical function; ④ mechanical ventilation time; ⑤ length of ICU stay; ⑥ length of hospital stay; ⑦ ICU mortality; ⑧ ICU-AW incidence; ⑨ muscle thickness; ⑩ adverse events.

6MWD = 6 minutes walking distance, ADL = activities of daily living, BI = Barthel Index, CAM-ICU = confusion assessment method for the intensive care unit, COPD = chronic obstructive pulmonary disease, E/C = experimental/control, FSS-ICU = Functional Status Score for the intensive care unit, IBC = in-bed cycling, ICU = intensive care unit, ICU-AW = ICU-acquired weakness, IMS = ICU mobilization score, MES = muscle electrical stimulation, MRC = Medical Research Council, MV = mechanical ventilation, NR = not report, RR = routine rehabilitation, SOMS = Surgical ICU Optimal Mobilization Score.

### 3.2. Study characteristics

The included studies originated from various countries, with a notable number from China,^[13,16,46,51,53,56–58,60,61]^ Brazil (n = 6),^[10,39,41,43,44,54,55]^ Australia (n = 2),^[9,19]^ and Belgium (n = 2).^[17,38]^ Additionally, 1 study each originated from Canada, Switzerland, the Czech Republic, and France.^[11,18,22,48]^ The sample sizes ranged from 16 to 360 participants, with a significant portion of the studies (n = 12) involving more than 100 cases.^[9,11,18,20,22,58,51,46,55,50,52,59]^ The total participant count across all studies was 3781. These studies covered a broad spectrum of ICU admissions reasons, including sepsis, severe pneumonia, postoperative conditions, respiratory failure, myocardial infarction, and postoperative abdominal surgery. Study sites spanned cardiovascular, medical, surgical and mixed ICUs, reflecting patient diversity, and representativeness. Intervention and control details are summarized in Table 1.

### 3.3. Assessment of risk of bias

**Figure 2.**
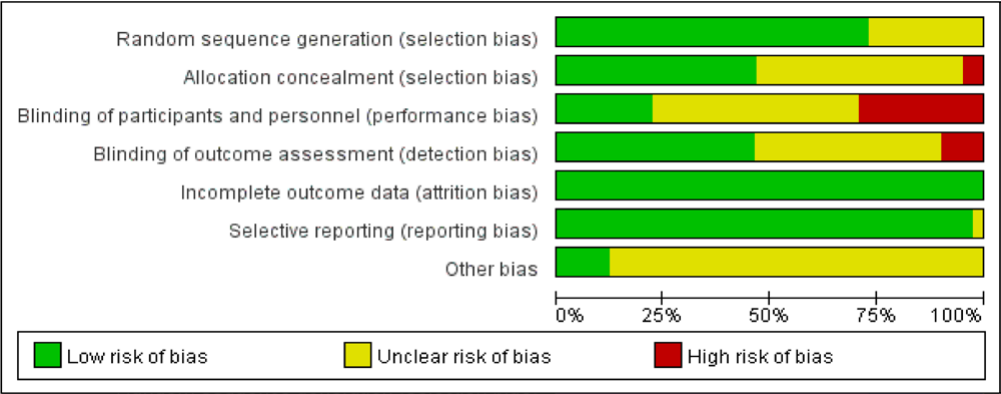
Overall risk of bias of the included studies. Risk of bias was assessed using the Cochrane Risk of Bias tool across standard domains.

Most trials reported adequate random sequence generation. Allocation concealment was frequently low-risk but often unclear, while blinding of participants/personnel was commonly high risk or unfeasible given the nature of rehabilitation. Outcome-assessor blinding was variably reported (often low risk, with several unclear). Attrition/data integrity and selective reporting were generally low risk, whereas “other bias” was usually unclear. Overall, performance bias and, to a lesser extent, concealment issues constituted the principal risks across studies. The methodological specifics are detailed in Table 2. Figure 2 presents the overall risk of bias for the included studies.

**Table 2:** Quality evaluation of RCT studies.

References	Random method	Allocation concealment	Blind methods		Data integrity	Selective reporting	Other bias
			For intervention subjects of intervener	For outcome surveyor			
Zhang et al^[13]^	Low-risk bias	High-risk bias	High-risk bias	Low-risk bias	Low-risk bias	Low-risk bias	Unclear
Soto et al^[14]^	Low-risk bias	Low-risk bias	Low-risk bias	Unclear	Low-risk bias	Low-risk bias	Unclear
Lorenz et al^[36]^	Low-risk bias	Low-risk bias	High-risk bias	Low-risk bias	Low-risk bias	Low-risk bias	Unclear
Kho et al^[20]^	Low-risk bias	Low-risk bias	High-risk bias	High-risk bias	Low-risk bias	Low-risk bias	Low-risk bias
Wu (2024)	Low-risk bias	High-risk bias	Unclear	Unclear	Low-risk bias	Low-risk bias	Unclear
Ahmad et al^[21]^	Low-risk bias	Low-risk bias	High-risk bias	High-risk bias	Low-risk bias	Low-risk bias	Unclear
Zhou et al^[60]^	Low-risk bias	High-risk bias	High-risk bias	Unclear	Low-risk bias	Low-risk bias	Unclear
Maca et al^[15]^	Low-risk bias	Low-risk bias	High-risk bias	Unclear	Low-risk bias	Low-risk bias	Unclear
Lin et al^[16]^	Low-risk bias	Low-risk bias	Low-risk bias	Low-risk bias	Low-risk bias	Low-risk bias	Unclear
Bano et al^[59]^	Low-risk bias	Low-risk bias	Low-risk bias	Unclear	Low-risk bias	Low-risk bias	Low-risk bias
Yu et al^[58]^	Low-risk bias	Unclear	High-risk bias	Unclear	Low-risk bias	Low-risk bias	Unclear
Qie et al^[57]^	Low-risk bias	Unclear	Unclear	Unclear	Low-risk bias	Low-risk bias	Unclear
Deng et al^[56]^	Low-risk bias	Low-risk bias	High-risk bias	Low-risk bias	Low-risk bias	Low-risk bias	Unclear
Waldauf et al^[22]^	Low-risk bias	Unclear	Unclear	Low-risk bias	Low-risk bias	Low-risk bias	Low-risk bias
De Azevedo et al^[55]^	Low-risk bias	Low-risk bias	High-risk bias	Low-risk bias	Low-risk bias	Low-risk bias	Unclear
Ribeiro et al^[54]^	Low-risk bias	Low-risk bias	High-risk bias	High-risk bias	Low-risk bias	Low-risk bias	Unclear
Berney et al^[9]^	Low-risk bias	Low-risk bias	Unclear	Low-risk bias	Low-risk bias	Low-risk bias	Low-risk bias
Schujmann et al^[52]^	Low-risk bias	Low-risk bias	High-risk bias	Low-risk bias	Low-risk bias	Low-risk bias	Unclear
Yu et al^[51]^	Low-risk bias	Unclear	Unclear	Low-risk bias	Low-risk bias	Low-risk bias	Unclear
Nickels et al^[19]^	Low-risk bias	Low-risk bias	Low-risk bias	Low-risk bias	Low-risk bias	Low-risk bias	Unclear
Ji et al^[53]^	Low-risk bias	Low-risk bias	Unclear	Unclear	Low-risk bias	Low-risk bias	Unclear
Gama et al^[50]^	Low-risk bias	Low-risk bias	Unclear	Low-risk bias	Low-risk bias	Low-risk bias	Unclear
Seo et al^[49]^	Unclear	Unclear	Unclear	Unclear	Low-risk bias	Low-risk bias	Unclear
Kho et al^[48]^	Low-risk bias	Low-risk bias	Low-risk bias	Unclear	Low-risk bias	Low-risk bias	Unclear
Carvalho 2019	Low-risk bias	Unclear	High-risk bias	Low-risk bias	Low-risk bias	Low-risk bias	Unclear
Ji (2019)	Unclear	Unclear	Unclear	Unclear	Low-risk bias	Low-risk bias	Unclear
Wu (2018)	Low-risk bias	Unclear	Unclear	Unclear	Low-risk bias	Low-risk bias	Unclear
Hickmann et al^[17]^	Unclear	Unclear	Low-risk bias	Low-risk bias	Low-risk bias	Low-risk bias	Unclear
Bianchi et al^[10]^	Low-risk bias	Low-risk bias	High-risk bias	Low-risk bias	Low-risk bias	Low-risk bias	Unclear
Eggmann et al^[18]^	Unclear	Low-risk bias	Low-risk bias	Low-risk bias	Low-risk bias	Low-risk bias	Unclear
Fossat et al^[11]^	Low-risk bias	Unclear	Low-risk bias	Low-risk bias	Low-risk bias	Low-risk bias	Low-risk bias
Zhu et al^[45]^	Low-risk bias	Unclear	High-risk bias	Unclear	Low-risk bias	Low-risk bias	Unclear
Dou et al^[46]^	Low-risk bias	Unclear	Unclear	Unclear	Low-risk bias	Low-risk bias	Unclear
França et al^[44]^	Unclear	Unclear	Low-risk bias	Unclear	Low-risk bias	Low-risk bias	Unclear
Machado et al^[43]^	Low-risk bias	Unclear	Unclear	Low-risk bias	Low-risk bias	Low-risk bias	Unclear
Borges et al^[42]^	Unclear	Unclear	Unclear	Unclear	Low-risk bias	Low-risk bias	Unclear
Coutinho et al^[41]^	Unclear	Unclear	Low-risk bias	Unclear	Low-risk bias	Low-risk bias	Unclear
Kayambu et al^[40]^	Low-risk bias	Unclear	High-risk bias	Low-risk bias	Low-risk bias	Low-risk bias	Unclear
Dantas et al^[39]^	Unclear	Unclear	Low-risk bias	Low-risk bias	Low-risk bias	Low-risk bias	Unclear
Burtin et al^[38]^	Low-risk bias	Low-risk bias	Unclear	Low-risk bias	Low-risk bias	Low-risk bias	Unclear
Nava^37]^,	Low-risk bias	Unclear	Unclear	Unclear	Low-risk bias	Low-risk bias	Unclear

RCT = randomized controlled trial.

### 3.4. Results of the meta-analysis

Meta-analysis indicates that adding IBC to routine rehabilitation increases muscle strength, reduces ICU-acquired weakness and delirium, and shortens the duration of MV and ICU stay, without increasing ICU mortality or serious exercise-related adverse events. A summary of findings is provided in Table 3.

**Table 3:** Results of meta-analysis.

Outcome indicators	Number of studies	*P*	*I* ^2^	Meta-analytic results		*P*	Sensitivity test results (change the model)		
				SMD	95% CI		SMD	95% CI	*P*
MRC score	12	<.001	93%	0.53	(0.01 to 1.05)	.04	0.29	(0.16 to 0.42)	<.001
6MWD	7	<.001	91%	0.80	(0.14 to 1.45)	.02	0.50	(0.33 to 0.70)	<.001
Handgrip strength	5	.27	23%	0.09	(0.02 to 0.36)	.03	0.20	(0.00 to 0.40)	.05
ADL score	10	<.001	88%	1.06	(0.66 to 1.46)	<.001	0.98	(0.84 to 1.12)	<.001
MV time	25	<.001	79%	-0.28	(-0.47 to −0.99)	.001	-0.29	(-0.38 to −0.21)	<.001
ICU stay	32	<.001	86%	-0.32	(-0.53 to −0.12)	.002	-0.34	(-0.41 to −0.26)	<.001
Hospital stay	21	<.001	74%	-0.22	(-0.40 to -0.03)	.02	-0.16	(-0.25 to -0,07)	.0005
ICU-AW incidence	9	.11	39%	0.49	(0.37 to 0.67)	<.001	0.47	(0.32 to 0.70)	.0003
Delirium incidence	9	.01	60%	0.50	(0.33 to 0.77)	.001	0.56	(0.44 to 0.0.71)	<.001
ICU mortality	18	.31	12%	0.95	(0.76 to 1.19)	.65	0.95	(0.73 to 1.23)	.69
Adverse events	11	.07	41%	0.77	(0.53 to 1.14)	.20	0.79	(0.41 to 1.52)	.48

6MWD = 6-minute walking distance, ADL = activities of daily living, CI = confidence interval, ICU = intensive care unit, ICU-AW = ICU-acquired weakness, MRC = Medical Research Council, MV = mechanical ventilation, SMD = standardized mean difference.

#### 3.4.1. MRC score

**Figure 3.**
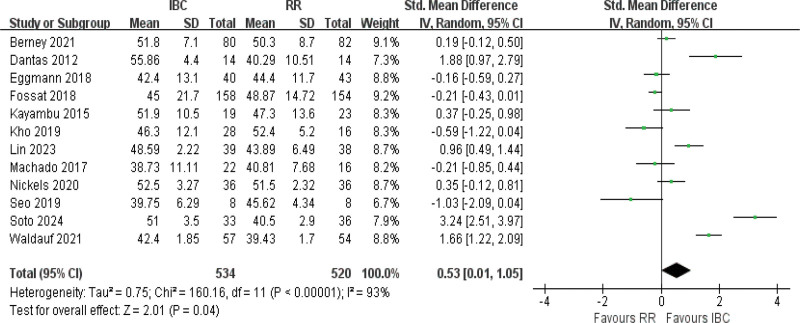
Forest plot of MRC score. Effect sizes are presented as standardized mean differences with 95% confidence intervals. MRC = Medical Research Council.

The analysis of MRC score pooled data from 12 studies,^[9,11,14,16,18,19,22,43,48,49,40]^ revealing high heterogeneity (*I*^2^ = 93%, *P* *<* .001). Across the included trials, the mean MRC score at ICU discharge was approximately 47.1 ± 8.2 in the IBC group and 43.3 ± 8.5 in the control group, yielding a weighted mean difference of about 3.8 points. The results showed that significant increase in MRC score among patients undergoing IBC (SMD = 0.54, 95% CI [0.01–1.05], *P* = .04; Fig. 3; very low certainty).

#### 3.4.2. 6MWD

**Figure 4.**
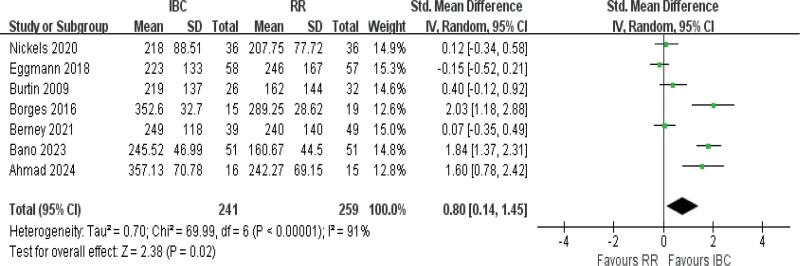
Forest plot of 6-minute walking distance (6MWD). Standardized mean differences with 95% confidence intervals are shown.

Data from 7 studies were analyzed,^[9,18,19,21,38,59,42]^ showing substantial heterogeneity (*I*^2^ = 91%, *P* *<* .001). The random-effects model did demonstrate significant differences in 6MWD for patients engaged in IBC compared to those in control groups (SMD = 0.80, 95% CI [0.14–1.45], *P* = .02; Fig. 4;very low certainty), with an average absolute improvement of approximately 45 to 60 m in 6MWD favoring the IBC group.

#### 3.4.3. BI

**Figure 5.**
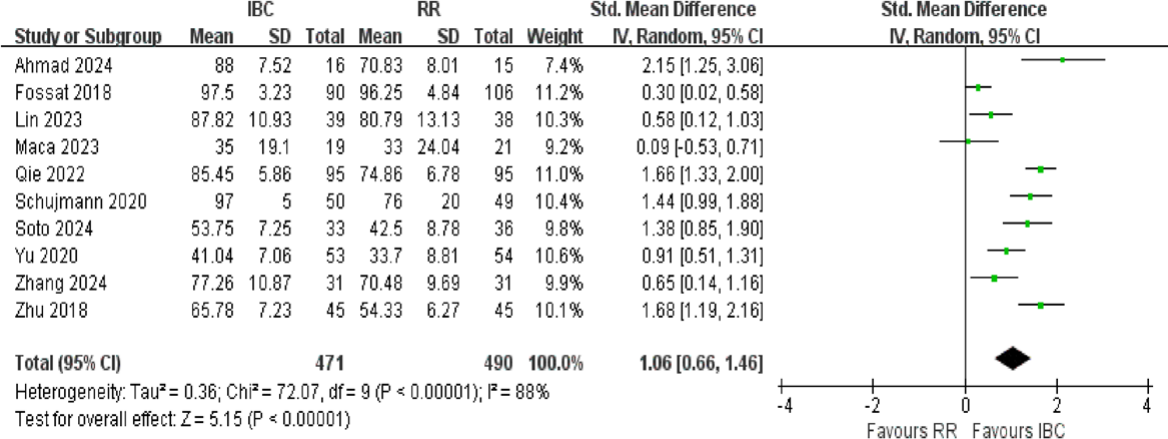
Forest plot of activities of daily living assessed by the Barthel Index. Effect estimates are expressed as standardized mean differences with 95% confidence intervals.

The ADL score was assessed by BI in ten studies,^[11,13–16,21,57,51,52,45]^ with results indicating high heterogeneity (*I*^2^ = 88%, *P* *<* .001). The results did demonstrate differences between the 2 groups (SMD = 1.06, 95% CI [0.66–1.46], *P* *<* .001; Fig. 5; moderate certainty), with an average absolute increase of approximately 10 to 11 points in the BI among patients receiving IBC compared with controls.

#### 3.4.4. Handgrip strength

**Figure 6.**
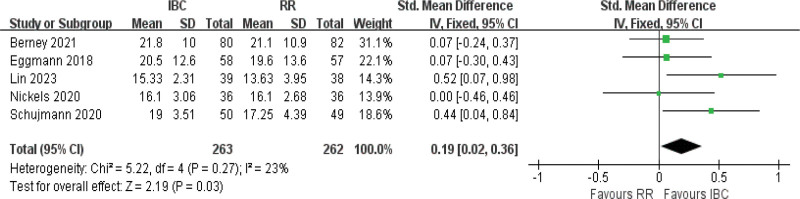
Forest plot of handgrip strength. Standardized mean differences with 95% confidence intervals are presented.

Analysis of 5 studies^[9,16,18,19,52]^ showed low heterogeneity (*I^2^* = 23%, *P* = .27) and revealed a significant improvement in handgrip strength (SMD = 0.19, 95% CI [0.02–0.36], *P = *.03; Fig. 6; moderate certainty), highlighting a potential benefit of IBC on handgrip strength. The IBC group showed a sample size-weighted mean improvement of +0.99 (range: 0.00–1.75; median: +0.90) versus controls.

#### 3.4.5. MV time

**Figure 7.**
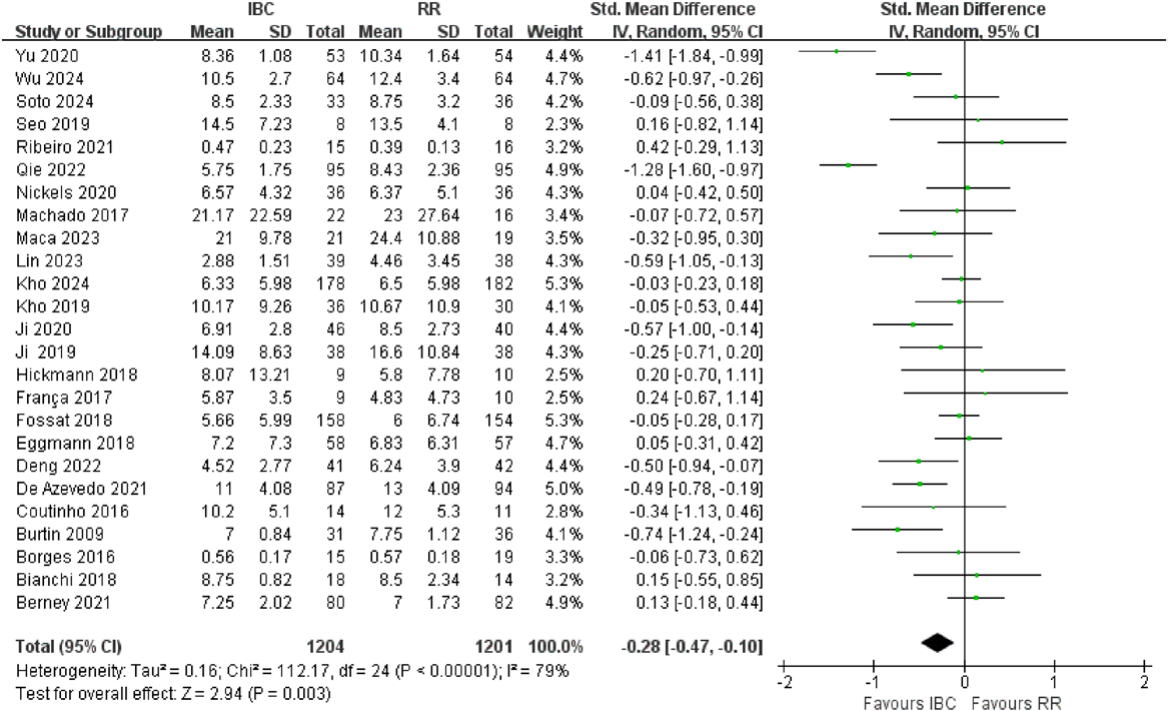
Forest plot of duration of mechanical ventilation. Effect sizes are expressed as standardized mean differences with 95% confidence intervals.

MV time, derived from 25 studies,^[12,14–17,19,61,51,53,56,57,54,49,40]^ showed high heterogeneity (*I*^2^ = 79%, *P* *<* .001) and demonstrated a significant reduction in MV time (SMD = −0.28, 95% CI [−0.47 to −0.10], *P* = .003; Fig. 7; very low certainty), corresponding to an average absolute reduction of approximately 1.4 days (median 1.3 days) in MV duration for patients receiving IBC.

#### 3.4.6. ICU-LOS

**Figure 8.**
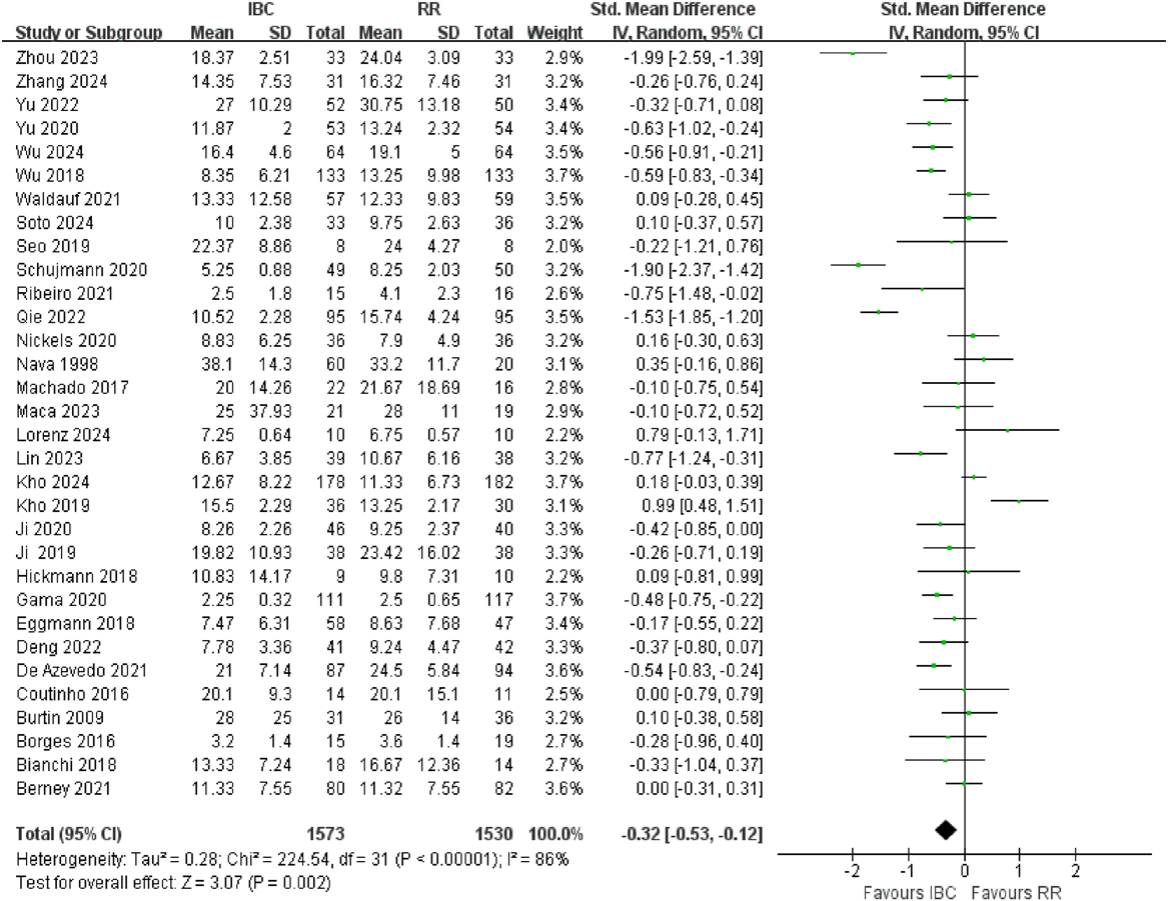
Forest plot of ICU length of stay. Standardized mean differences with 95% confidence intervals are shown. ICU = intensive care unit.

Thirty-two articles were included for ICU stay.^[9,12–16,19,20,22,51,53,56–58,60,61,54,55,48,50,52,49,40,45,62]^ The results of the *Q* test indicated high heterogeneity with *I*^2^ = 86% and *P* < .001, the results are significant (SMD = −0.32, 95% CI (−0.53 to −0.12), *P* = .0002; Fig. 8; very low certainty), suggesting that the IBC group had a mean 1.5 to 2 day reduction in ICU-LOS versus controls (range: 0.3–3.6 days).

#### 3.4.7. Hospital stay

**Figure 9.**
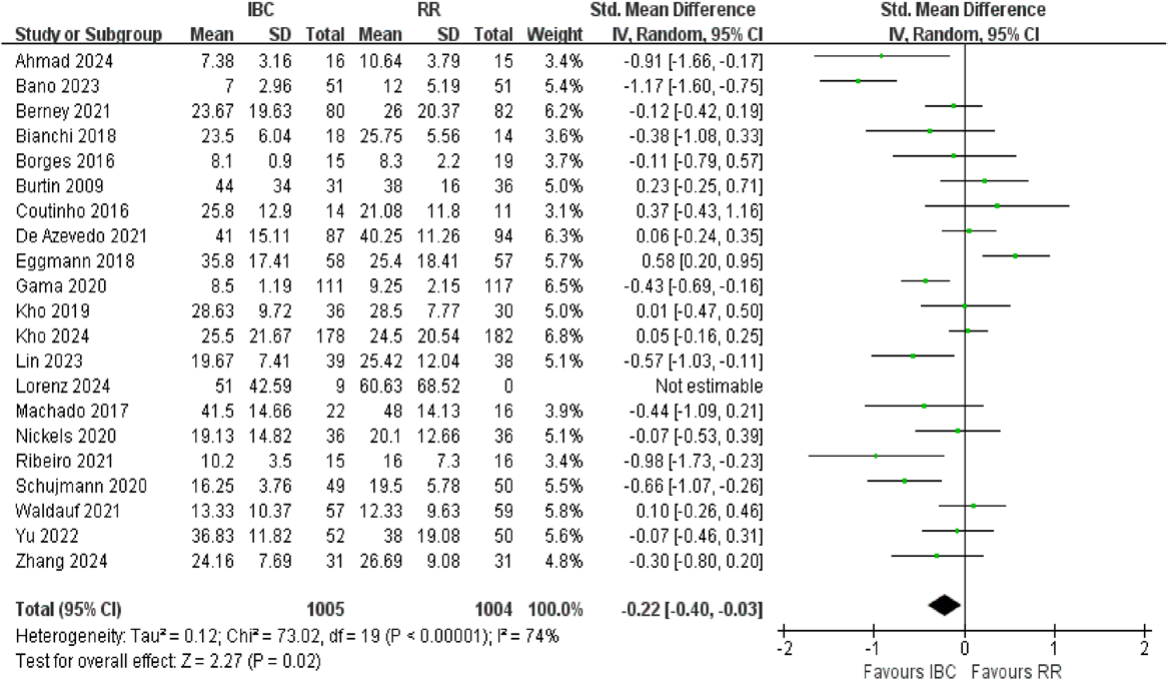
Forest plot of hospital length of stay. Effect estimates are presented as standardized mean differences with 95% confidence intervals.

Data from 21 studies^[10,13,19–22,36,58,43,41,38,48,50,54,55,59,42,37]^ highlighted moderate heterogeneity (*I*^2^ = 74%, *P* < .001) and a significant reduction in the duration of hospital stay (SMD = −0.22, 95% CI [−0.40 to −0.03], *P* = .02; Fig. 9; low certainty). On an absolute scale, the between-group difference corresponded to an average reduction of approximately 1.5 to 2 days (range 0.5–4.5 days) in total hospital length of stay among patients receiving IBC.

#### 3.4.8. ICU-AW incidence

**Figure 10.**
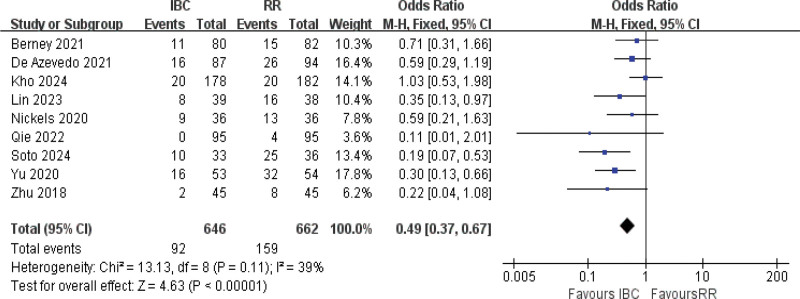
Forest plot of ICU-acquired weakness incidence. Odds ratios with 95% confidence intervals are presented. ICU = intensive care unit.

Nine studies were included in the meta-analysis.^[9,14,16,20,58,57,51,55,45]^ The results of the *Q* test indicated fewer heterogeneity with *I*^2^ = 39% and *P* = .11. Employing the fixed-effects model analysis (Fig. 10), the results showed statistical significance between 2 groups (OR = 0.49, 95% CI [0.37–0.67], *P* <.001; moderate certainty). ICU-AW incidence: 14.2% (92/646) with IBC versus 24.0% (159/662) in controls, demonstrating an absolute risk reduction of 9.8 percentage points.

#### 3.4.9. Delirium incidence

**Figure 11.**
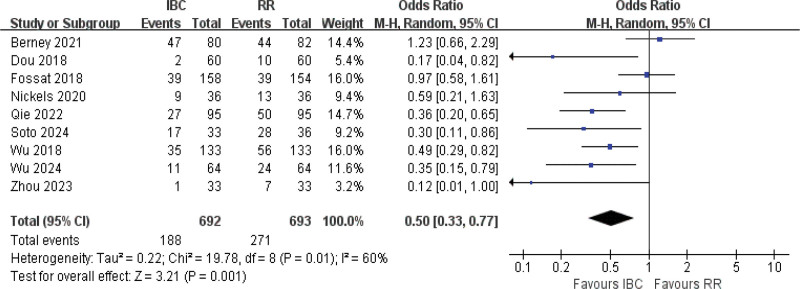
Forest plot of delirium incidence. Odds ratios with 95% confidence intervals are shown.

Nine studies were included in the meta-analysis.^[9,11,14,19,61,60,57,46,62]^ The results of the *Q* test indicated moderate heterogeneity with *I*^2^ = 60% and *P* = .01. Employing the random-effects model analysis (Fig. 11), the results showed statistical significance between 2 groups (OR = 0.50, 95% CI [0.33–0.77], *P* = .001; low certainty). Delirium occurred in 27.2% of IBC patients versus 39.1% of controls, demonstrating an absolute risk reduction of 11.9 percentage points and a relative risk reduction of 49.9%.

#### 3.4.10. ICU mortality and adverse events

**Figure 12.**
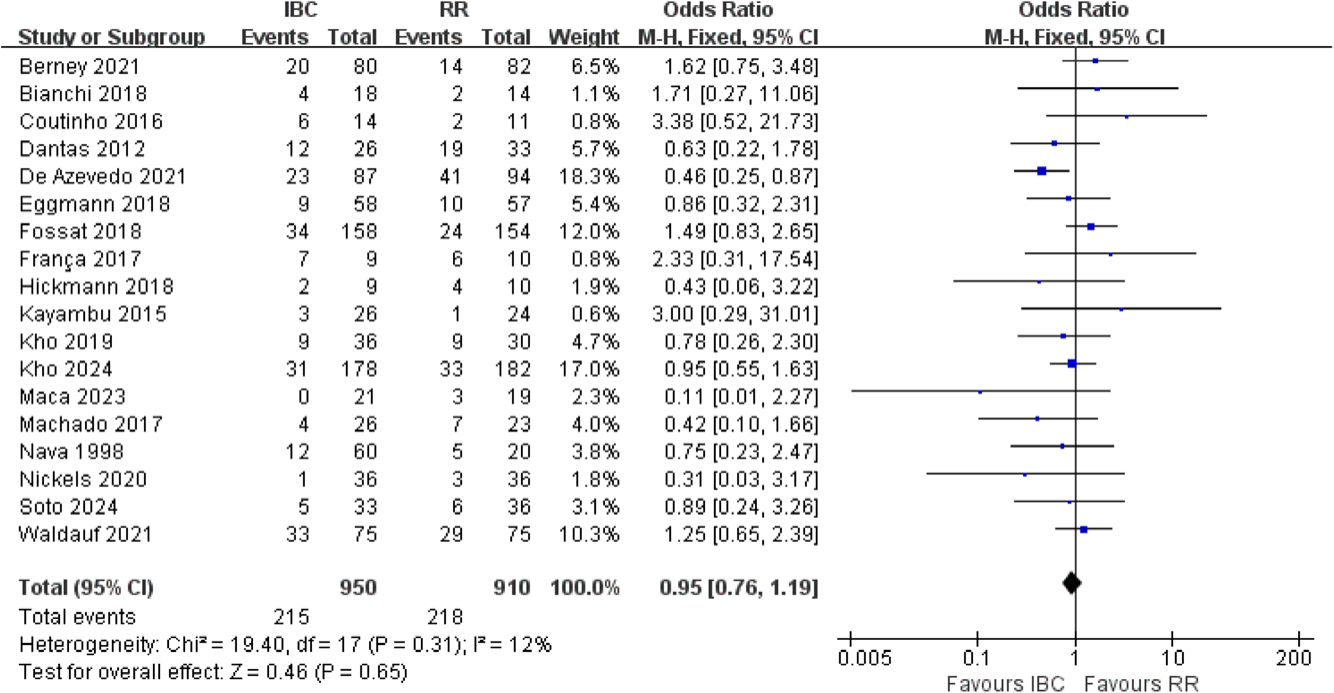
Forest plot of ICU mortality. Odds ratios with 95% confidence intervals are presented. ICU = intensive care unit.

**Figure 13.**
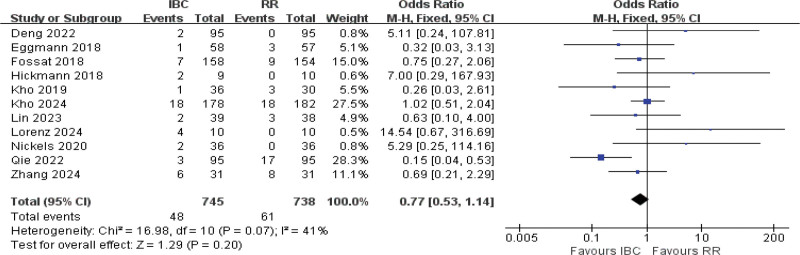
Forest plot of adverse events. Odds ratios with 95% confidence intervals are shown.

Eighteen studies were included in the meta-analysis^[11,14,15,17–20,22,39,41,43,44,55,48,40,37]^ for ICU mortality and eleven studies included for adverse events.^[11,13,16–20,36,57,56,48]^ Employing the fixed-effects model analysis, the results showed no statistical significance between 2 groups (OR = 0.95, 95% CI [0.76–1.19], *P* = .65; Fig. 12; moderate certainty) of ICU mortality and (OR = 0.77, 95% CI [0.53–1.14], *P* = .20; Fig. 13; low certainty) of adverse events. These results underscore the safety of IBC.

### 3.5. Sensitivity and publication bias analysis

**Figure 14.**
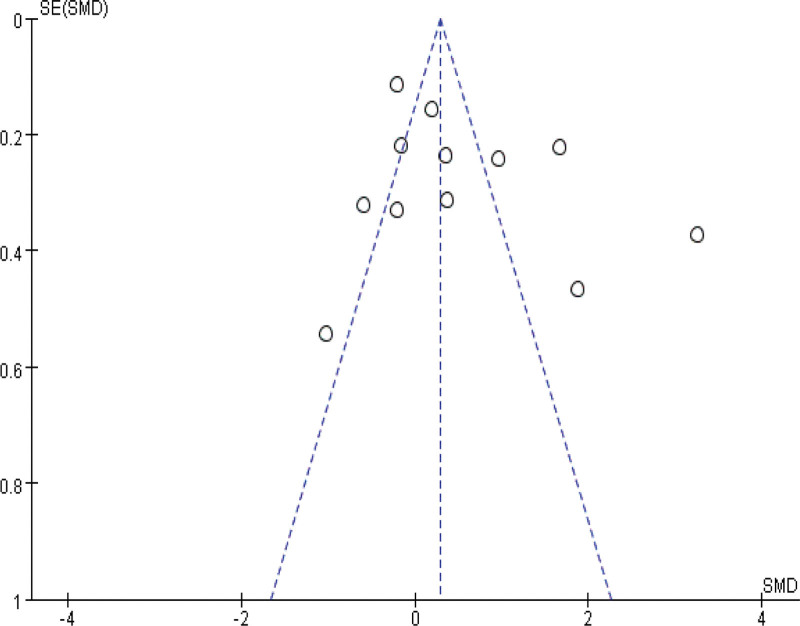
Funnel plot of MRC score. Each dot represents an individual study. MRC = Medical Research Council.

**Figure 15.**
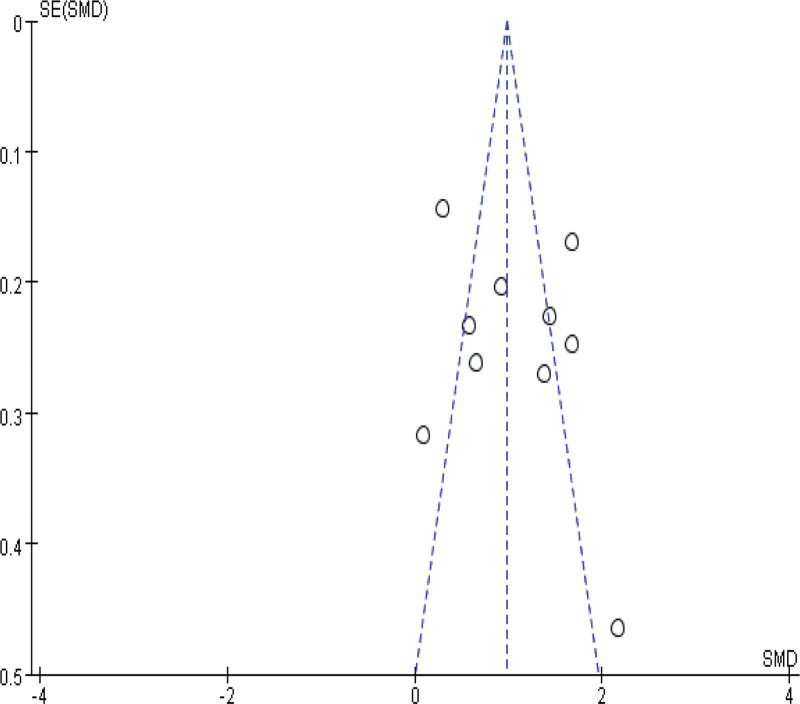
Funnel plot of Barthel Index. Each dot represents an individual study.

**Figure 16.**
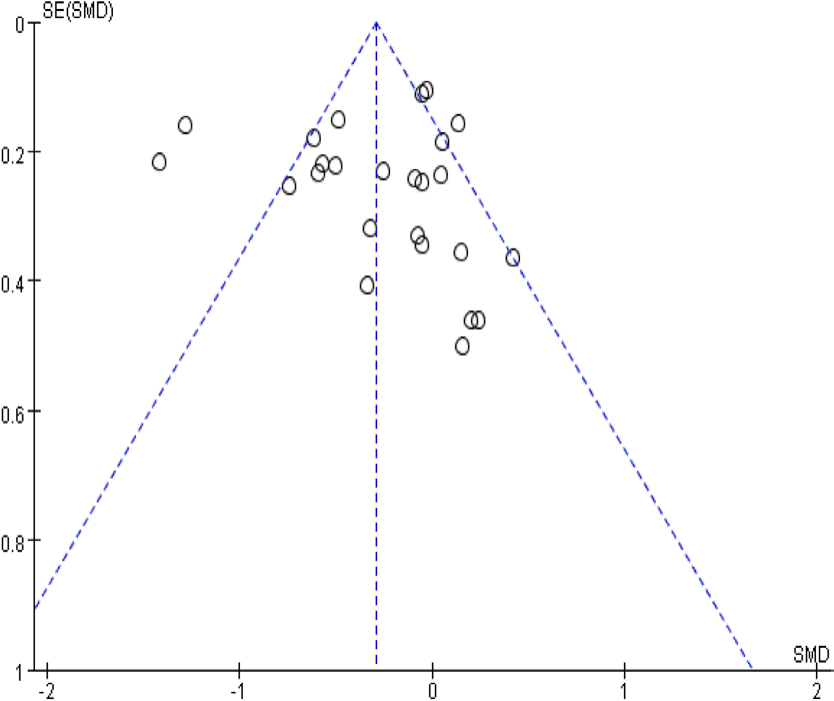
Funnel plot of mechanical ventilation time. Each dot represents an individual study.

**Figure 17.**
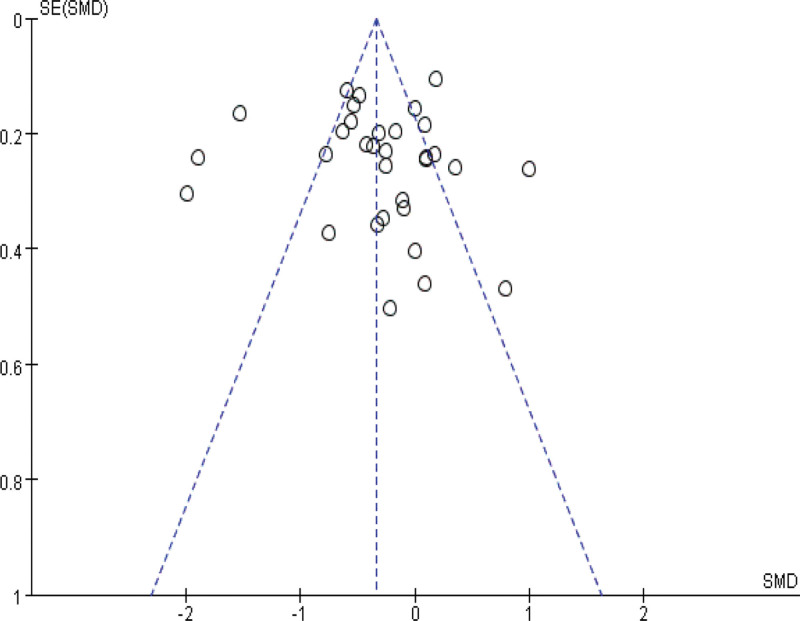
Funnel plot of ICU length of stay. Each dot represents an individual study. ICU = intensive care unit.

**Figure 18.**
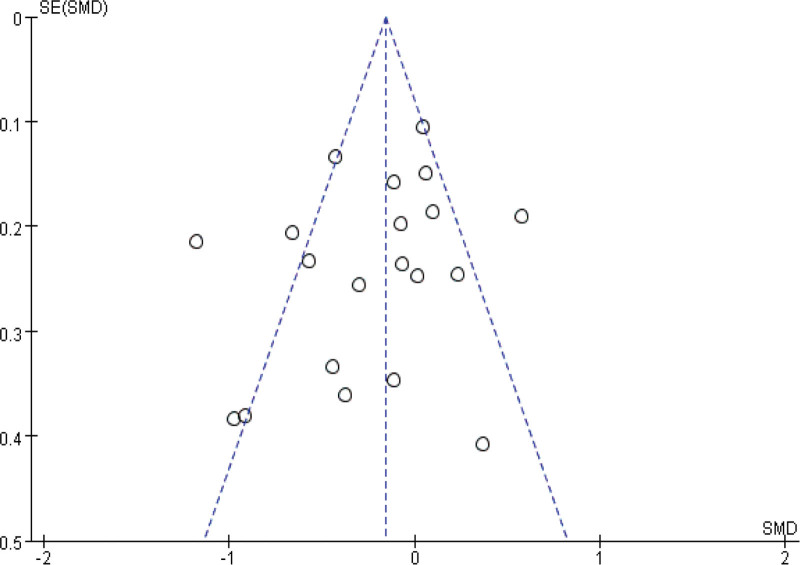
Funnel plot of hospital length of stay. Each dot represents an individual study.

**Figure 19.**
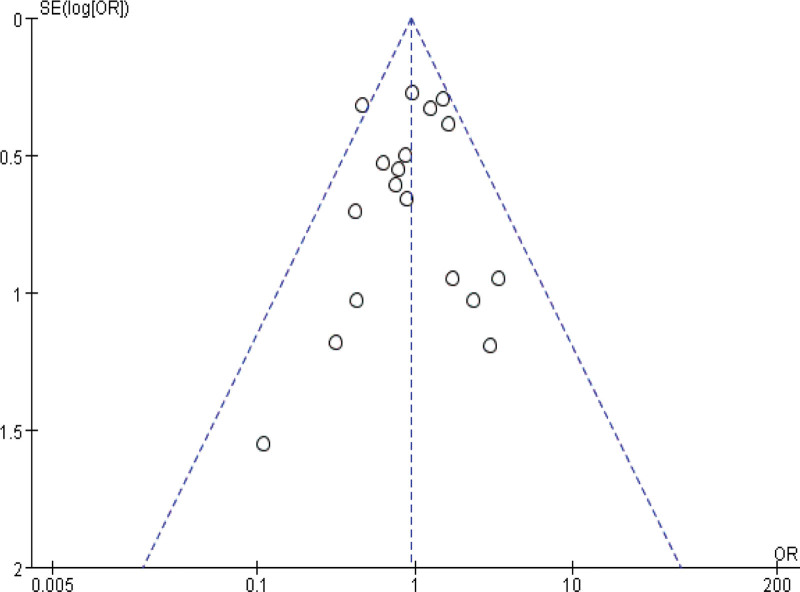
Funnel plot of ICU mortality. Each dot represents an individual study. ICU = intensive care unit.

**Figure 20.**
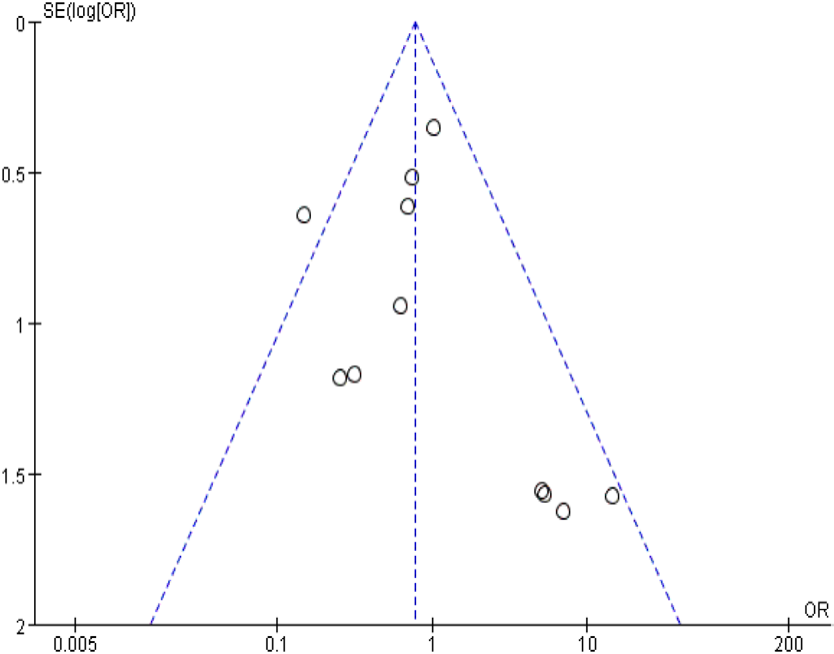
Funnel plot of adverse events. Each dot represents an individual study.

Sensitivity analyses were performed by modifying the analytical models across various clinical indicators. These analyses did not reveal significant changes, underlining the robustness of our findings. These findings support the potential of IBC to enhance physical function in ICU patients, shorten ICU length of stay, and reduce ICU complications. The specific outcomes of the sensitivity analyses are detailed in Table 3. Funnel plot analysis reveals that, apart from mild asymmetry in BI, length of hospital stay, and incidence of adverse events, all other outcomes show no evident publication bias. See Figures 14–20 for specific details.

### 3.6. Grading of evidence for meta-analysis results

The quality of evidence for each outcome indicator was graded according to the Grading of Recommendations Assessment, Development and Evaluation system, with classifications ranging from moderate to very low. The consensus between 2 independent reviewers on the evidence grading reinforces the credibility of the grading process. The outcomes, summarized in Table 4, highlight the need for further high-quality research to substantiate the findings from existing studies. This grading underscores the importance of treating current conclusions with caution and reflects the ongoing need for rigorous studies to fully understand the impacts of IBC exercise in ICU settings.

**Table 4:** Grading of Recommendations Assessment, Development and Evaluation evidence profile in the meta-analysis of IBC.

Items	Certainly assessment							No. of patients	Effect	Quality
Outcomes	No. of studies	Design	Risk of bias	Inconsistency	Indirectness	Imprecision	Other considerations	Total	Relative/absolute (95% CI)
MRC score	12	Randomized	Serious*	Serious†	Serious‡	Not serious	None	1054	SMD 0.29 95% CI (0.16 to 0.42)	⊕○○○ Very low
6MWD	7	Randomized	Serious*	Serious†	Not serious	Not serious	Serious§	500	SMD 0.80 95% CI (0.14 to 1.45)	⊕○○○ Very Low
Handgrip strength	5	Randomized	Serious*	Not serious	Not serious	Not serious	None	525	SMD 0.19 95% CI (0.02 to 0.36)	⊕⊕⊕○ Moderate
ADL score	10	Randomized	Serious*	Serious†	Not serious	Not serious	None	961	SMD 1.06 95% CI (0.66 to 1.46)	⊕⊕○○ Low
MV time	25	Randomized	Serious*	Serious†	Not serious	Not serious	None	2405	SMD 0.29 95% CI (0.38 to 0.21)	⊕○○○ Very low
ICU stay	31	Randomized	Serious*	Serious†	Not serious	Not serious	None	3023	SMD −0.34 95% CI (-0.55 to -0.14)	⊕⊕○○ Low
Hospital stay	21	Randomized	Serious*	Not serious	Not serious	Serious‡	None	2009	SMD −0.14 95% CI (-0.32 to -0.03)	⊕⊕○○ Low
ICU-AW incidence	9	Randomized	Serious*	Not serious	Not serious	Not serious	None	1308	OR 0.49 95% CI (0.37 to 0.67)	⊕⊕⊕○ Moderate
Delirium incidence	9	Randomized	Serious*	Not serious	Not serious	Not serious	Serious§	1385	OR 0.50 95% CI (0.33 to 0.77)	⊕⊕○○ Low
ICU mortality	18	Randomized	Serious*	Not serious	Not serious	Not serious	None	1780	OR 0.96 95% CI (0.76 to 1.20)	⊕⊕⊕○ Moderate
Adverse events	11	Randomized	Serious*	Not serious	Serious‡	Not serious	None	1483	OR 77 95% CI (0.0.53 to 1.24)	⊕⊕○○ Low

6MWD = 6-minute walking distance, ADL = activities of daily living, CI = confidence interval, IBC = in-bed cycling, ICU = intensive care unit, ICU-AW = ICU-acquired weakness, MRC = Medical Research Council, MV = mechanical ventilation, SMD = standardized mean difference.

* Lack of allocation concealment or blinding.

† *I*^2^ > 75%, PI crosses both harm and benefit.

‡ Population risks differed significantly across studies.

§ The funnel plot is asymmetric.

## 4. Discussion

### 4.1. Main findings and heterogeneity analysis

This updated study conducted a systematic review and meta-analysis of the recent RCTs on IBC in adult ICUs to evaluate its impact on clinical outcomes. Across 41 studies, with diverse populations, regions, diagnoses, and intervention protocols, we focused on outcomes at ICU discharge to minimize post-ICU confounding and program interruption. The principal finding is that, compared with standard ICU rehabilitation alone, adding progressive IBC improves muscle strength scores, lowers ICU-acquired weakness and delirium rates, shortens MV duration and ICU length of stay, and does not increase ICU mortality or exercise-related adverse events.

Compared with 2 prior reviews on similar topics,^[63,64]^ this study incorporated additional function-related outcomes: delirium incidence, handgrip strength, and the BI, and synthesized a larger evidence base (40 studies), enabling a more comprehensive appraisal of IBC effects on ICU patients’ muscle strength and physical function. In meta-analytic terms, our estimates for MV duration, ICU-LOS, total hospital length of stay, ICU mortality, 6MWD, and adverse event rates align with O’Grady and Rocío, whereas findings for MRC scores and ICU-acquired weakness contrast with Rocío,^[63]^ likely reflecting greater statistical power from a larger pool of studies. Unlike the previous 2 studies, we did not meta-analyze quality of life owing to its complex determinants after ICU discharge and heterogeneity of assessment tools. Overall, this updated meta-analysis highlights the positive impact of IBC on acute neurocognitive outcomes (delirium), physical function, and ventilation-related endpoints in ICU populations.

However, several analyses showed substantial heterogeneity (*I*^2^ > 70% in 6 outcomes, as shown in Table 3); many trials lacked robust randomization, allocation concealment or blinding, and most outcomes (≈60%) were graded as very low/low certainty. These limitations warrant cautious interpretation and further confirmatory research. High heterogeneity in MRC, BI, MVT, ICU-LOS, and total hospital stay likely reflects: substantial population variability, as participants spanned sepsis/septic shock, post-cardiac surgery, acute exacerbation of chronic obstructive pulmonary disease, abdominal surgery, aortic dissection, and COVID-19, with differing disease phases and baseline function shaping recovery trajectories; and diverse IBC prescriptions: active versus passive cycling, with/without electrical stimulation, session frequency (1–2/day), duration (10–90 minutes), initiation (during intubation, post-extubation or on ICU admission) and treatment course (to extubation, 7 days or ICU transfer), yielding disparate outcomes (MVT, MRC, ICU stay). Finally, statistical work suggests heterogeneity is more readily detected as the number of included studies grows, even when pooled estimates remain relatively stable.^[65]^ Overall, heterogeneity appears population- and intervention-driven; standardizing prescriptions, harmonizing endpoints, and strengthening stratified and sensitivity analyses should improve consistency and interpretability.

### 4.2. The effect of IBC on muscle strength and ICU-AW

Evidence indicates that muscle mass can decline by up to 14% within the 1st ICU week, with atrophy progressing faster in mechanically ventilated patients.^[66,67]^ Preventing and treating ICU-AW is therefore a clinical priority, and IBC is 1 potential strategy.^[63]^ In this synthesis, 1 to 2 daily IBC sessions added to usual rehabilitation significantly improved MRC score, 6MWD, ADL score, handgrip strength, and ICU-AW rates, consistent with O’Grady et al,^[64]^ but contrasting with Rocío et al.^[63]^ The analysis included a larger sample size, which may account for the differing conclusions. The benefits of IBC likely reflect multifactorial biology: early critical illness drives rapid skeletal-muscle wasting and proteolysis, notably quadriceps thinning in week 1.^[68]^ Active or passive leg cycle ergometry provides repeated muscle-pump contractions that enhance limb perfusion, suppress proteolysis, and sustain fiber recruitment, thereby mitigating ICU-AW.^[69]^ Additionally, the rhythmic mechanical and sensory stimulation from IBC may attenuate systemic inflammation and oxidative stress, preserve mitochondrial function and neuromuscular junction transmission, and thus improve strength, mobility, and the risk profile for ICU-AW.^[2]^ Notably, apart from ICU-AW, the certainty of evidence for related outcomes was generally low, underscoring the need for high-quality confirmatory trials.

To extend the discussion, it is crucial to contextualize these findings within the broader landscape of ICU rehabilitation. Our results suggest that IBC is not merely an additive therapy but may act synergistically with routine rehabilitation components. IBC delivers doseable muscle-pump and cardiopulmonary stimulation to bridge the bed-rest “activity gap”; integrated with early stratification, graded mobilization and respiratory training in routine ICU care, it safely improves function and shortens ventilation. Future research should prioritize determining the optimal “dose” of IBC, including timing, intensity, and duration, across different patient subgroups, such as those with sepsis versus post-surgical states, where the pathophysiology of muscle wasting may differ. Moreover, while the physiological mechanisms are compelling, the relatively low certainty of evidence for outcomes other than ICU-AW incidence highlights a critical gap. This underscores the imperative for large-scale, high-quality randomized controlled trials that employ standardized, objective outcome measures (e.g., lower-limb ultrasonography and electromyography) and are powered to confirm not only efficacy but also the long-term functional benefits and safety of IBC in the heterogeneous ICU population.

### 4.3. The effect of IBC on ICU delirium and mortality

Delirium occurs in 30% to 60% of ICU patients, with a 1.52-fold higher risk in those on MV,^[70]^ and is associated with longer ICU stay and higher mortality.^[71]^ Although early mobilization is linked to lower delirium incidence,^[72]^ the effect of IBC remains debated.^[9,14,60]^ In this analysis, adding IBC to usual rehabilitation significantly reduced delirium without increasing ICU mortality or exercise-related adverse events; however, small samples and low-certainty evidence warrant caution. Potential mechanisms include greater daytime activity and reduced sedative exposure, with IBC (active or passive) enhancing wakefulness and sensory input, restoring circadian rhythms and sleep.^[73]^ When coordinated with light sedation and spontaneous breathing trials, IBC may lessen sedative use, facilitate weaning, and improve oxygenation and cerebral perfusion.^[18,74]^ Biologically, early mobilization centered on IBC may down-regulate interleukin-6/tumor necrosis factor-alpha and oxidative stress, up-regulate brain-derived neurotrophic factor, modulate microglial activation, and promote synaptic plasticity, thereby lowering delirium risk.^[75]^ Furthermore, ICU mortality and overall adverse event rates did not differ between groups; IBC-related events were 1.2%, chiefly transient blood-pressure or oxygen-saturation changes, supporting its safety.

Given the current low-certainty evidence, future studies should evaluate how different intensities and modalities of IBC affect delirium subtypes and duration in ICU patients over the longer term. Biomarker assays and neurophysiological monitoring are recommended to delineate mechanistic pathways, particularly effects on neuroinflammation, cerebral blood flow and brain network function. Research should also compare responses across key subgroups (e.g., older adults, sepsis, differing durations of MV) to advance personalized, precision delirium prevention. IBC is a promising, sustainable non-pharmacological rehabilitation strategy; future work should clarify mechanisms and enable systematic, individualized use.

### 4.4. The effect of IBC on MV time and ICU stay

Evidence on whether IBC shortens MV time and ICU length of stay remains mixed,^[20,36,52]^ likely reflecting patient complexity, variation in IBC prescriptions and differences in co-interventions.Despite high heterogeneity on *Q* tests, this meta-analysis indicates that IBC is associated with shorter MV and ICU-LOS, aligning with prior reports.^[63,64]^ Mechanistically, early critical illness entails rapid muscle loss and hypercatabolism; the rhythmic contractions induced by IBC may partly counterbalance proteolysis, preserving quadriceps thickness/cross-sectional area and mitochondrial–metabolic capacity, thereby enabling earlier mobilization and functional recovery.^[68]^ IBC may also enhance endurance of respiratory and peripheral muscles, facilitate secretion clearance, improve lung compliance and V/Q matching, and reduce the work of breathing, thereby improving readiness for weaning.^[76]^ In addition, cyclical active/passive contractions generate a “muscle-pump” effect that augments venous return and microvascular shear, supports organ perfusion and is associated with fewer complications (thrombosis, atelectasis, delirium), collectively compressing ICU and total hospital stay.^[69]^ Overall, with rigorous safety screening and individualized dosing, embedding IBC within standardized early mobilization pathways may expedite weaning and recovery while reducing resource use; definitive confirmation of long-term benefit and cost-effectiveness requires high-quality multicenter RCTs.

### 4.5. Clinical practice and research recommendations

In summary, as part of early mobilization, IBC may confer specific advantages in attenuating strength loss, enhancing functional independence, maintaining overall physical function, and reducing complications. We recommend embedding IBC in ICU early-activity pathways guided by the principles of “early initiation, stepwise progression, individualization and safety 1st.” Develop standardized IBC prescriptions and team training, with audit–feedback for quality improvement. Prioritize pilot implementation in high-risk units with anticipated prolonged ventilation, aligned with local resources and ethics, and conduct concurrent real-world cost-effectiveness evaluation.

Given methodological limitations and low certainty of existing evidence, future research should focus on the timing of IBC initiation and protocol standardization, accounting for diagnosis and IBC modality. In future large-sample studies, use a multimodal assessment (e.g., muscle strength scales, functional independence, imaging, muscle quantity and quality, inflammatory markers and neuromuscular activity) to quantify effects on muscle loss and atrophy. As IBC load is constrained by fitness and nutritional reserves, stratified and dose response analyses should test interactions between nutrition strategies (early enteral or parenteral, protein and energy targets and timing) and IBC, and their medium to long-term effects on function and ventilation outcomes. Furthermore, recognizing ICU illness complexity and volatility, trials should prospectively record at prespecified time points hemodynamics, sedative, and analgesic doses, cognition and mood, ventilatory status, IBC dosing and frequency, device settings and adherence to analyze interindividual variability, confounding, and their impact on IBC effectiveness.

### 4.6. Strengths and limitations

Strengths include a broad search across multiple languages, yielding a comprehensive view of IBC evidence in ICU settings, and the most up-to-date synthesis of diverse clinical outcomes to inform practice. Consistency and stability of pooled effects further support reliability. Limitations are: suboptimal randomization, allocation concealment and blinding in several trials, with possible overestimation of IBC effects; frequent use of composite interventions, hindering isolation of IBC’s independent impact; conversion of non-normal data to means/SDs, introducing modest imprecision; and insufficient studies with harmonized data to meta-analyze lower-limb muscle thickness. These factors temper the certainty of inference, though the findings still provide a valuable perspective on IBC’s potential benefits in critical-care rehabilitation.

## 5. Conclusion

When added to usual ICU rehabilitation and titrated to patient status, IBC is associated with improved muscle strength and limb function, lower rates of ICU-acquired weakness and delirium, and shorter durations of MV and ICU stay, without excess ICU mortality or exercise-related adverse events. IBC should be considered within a graded early-rehabilitation program, supported by consensus-based guidance on patient selection, initiation, dosing, frequency, and safety procedures. To more accurately evaluate the real-world effects of IBC in ICU patients and elucidate potential mechanisms, particularly in preventing muscle loss and strength decline, well-designed, adequately powered multicentre studies with comprehensive assessments are needed.

## Acknowledgments

We extend our heartfelt gratitude to all the dedicated authors who actively contributed to the data collection, analysis, and meticulous revisions throughout the course of this article’s development.

## Author contributions

**Data curation:** Ping Zhao, Deping Lü.

**Formal analysis:** Xihui Sun, Ping Zhao.

**Methodology:** Deping Lü, Ping Zhao, Xihui Sun.

**Validation:** Huineng Xiao, Min Feng.

**Writing – original draft:** Deping Lü, Huineng Xiao.
